# What Is the Role of Apelin regarding Cardiovascular Risk and Progression of Renal Disease in Type 2 Diabetic Patients with Diabetic Nephropathy?

**DOI:** 10.1155/2013/247649

**Published:** 2013-09-09

**Authors:** Ana Paula Silva, André Fragoso, Claudia Silva, Carla Viegas, Nelson Tavares, Patrícia Guilherme, Nélio Santos, Fátima Rato, Ana Camacho, Cidália Cavaco, Victor Pereira, Marilia Faísca, João Ataíde, Ilídio Jesus, Pedro Neves

**Affiliations:** ^1^Nephrology Department, Hospital Faro, Rua Leão Penedo, 8000-386 Faro, Portugal; ^2^Pathology Clinic, Hospital Faro, 8000-836 Faro, Portugal; ^3^Science of the Sea Department, University of Algarve, 8000-140 Faro, Portugal; ^4^Cardiology Department, Hospital Faro, 8000-386 Faro, Portugal; ^5^Pharmacology Department, Gnostic Laboratory, 8000-307 Faro, Portugal

## Abstract

*Aims*. To evaluate the association of different apelin levels with cardiovascular mortality, hospitalization, renal function, and cardiovascular risk factors in type 2 diabetic patients with mild to moderate CKD. *Methods*. An observational, prospective study involving 150 patients divided into groups according to baseline apelin levels: 1 ≤ 98 pg/mL, 2 = 98–328 pg/mL, and 3 ≥ 329 pg/mL. Baseline characteristics were analyzed and compared. Multivariate Cox regression was used to find out predictors of cardiovascular mortality, and multivariate logistic regression was used to find out predictors of hospitalization and disease progression. Simple linear regressions and Pearson correlations were used to investigate correlations between apelin and renal disease and cardiovascular risk factors. *Results*. Patients' survival at 83 months in groups 1, 2, and 3 was 39%, 40%, and 71.2%, respectively (*P* = 0.046). Apelin, age, and eGFR were independent predictors of mortality, and apelin, creatinine, eGFR, resistin, and visfatin were independent predictors of hospitalization. Apelin levels were negatively correlated with cardiovascular risk factors and positively correlated with eGFR. Patients with lower apelin levels were more likely to start a depurative technique. *Conclusions*. Apelin levels might have a significant clinical use as a marker/predictor of cardiovascular mortality and hospitalization or even as a therapeutic agent for CKD patients with cardiovascular disease.

## 1. Introduction

Cardiac diseases are independently associated with a deterioration of renal function and worsening of existing kidney disease. On the other hand, chronic kidney disease (CKD) is an independent risk factor for increased cardiovascular morbidity and mortality [1]. It has a complex pathogeneses, and traditional risk factors are not able to fully explain its high incidence and prevalence [2]. 

After many years of understanding as a simple fat storage, adipose tissue is nowadays considered an active endocrine organ with effects on other organs and systems [3]. Several substances produced by adipocytes—adipokines—have been identified, and they seem to play important roles in different physiological functions. They act at various levels, including the immune, endocrine, metabolic, and cardiovascular systems [4]. Among these substances, visfatin, resistin, and apelin might be implicated on the cardiorenal axis dysfunction [5].

Apelin, a circulating peptide produced and secreted by adipocytes, is a ligand for the APJ receptor [6]. Both apelin and APJ mRNA are abundantly expressed in a variety of tissues including kidneys and at various sites within the cardiovascular system [7]. Recent studies indicate that apelin signaling may be involved in the regulation of vascular tone, cardiac contractile function, and fluid balance [8].

Nowadays there are an increasing number of studies assessing apelin in an attempt to clarify its role as a cardiovascular disease (CVD) marker. Despite the notable interest in this relatively new adipokine, there are still many unknowns, and, in some cases, opposite results have been described. Furthermore, there is a lack of studies evaluating apelin on cardiovascular disease in patients with underlying renal failure. 

In the present study we evaluated the association of apelin levels with mortality and hospitalization events, as well as the relationship of apelin with renal function and cardiovascular risk factors, in a homogeneous population of type 2 diabetic patients with a diagnosis of mild to moderate CKD.

## 2. Material and Methods

An observational, prospective study involving 150 patients with type 2 diabetes mellitus was conducted in our outpatient diabetic nephropathy clinic from January 2005 to December 2011.

### 2.1. Subjects

Type 2 diabetic patients with a diagnosis of mild to moderate CKD (15 mL/min/1.73 m^2^ < eGFR ≤ 80 mL/min/1.73 m^2^) were eligible to participate in the study. The classification of diabetes was performed based on the guidelines from the American Diabetes Association [9]. 

The exclusion criteria were previous CVD—defined as a history of one or more of the following: nonfatal myocardial infarction, angina pectoris (stable or unstable), stroke or transient ischemic attacks, peripheral vascular disease or congestive heart failure, uncontrolled hypertension (BP ≥ 140/90 mmHg), albumin/creatinine ratio (UACR) > 500, eGFR ≤ 15 mL/min or > 80 mL/min, PTH ≥ 350 mmHg, phosphorus > 5.5, type 1 diabetes, nondiabetic renal disease, or neoplastic or infectious diseases.

All mortalities and hospitalization motivated by causes other than cardiovascular events were excluded. 

Patients were recruited on an outpatient nephrology consultation.

### 2.2. Follow-Up

Patients returned on a regular basis for in-person visits on nephrology consultation two to three times a year. The continuity of the follow-up of patients in the same institution is assured since in the Algarve region patients with renal disease are referred to Hospital de Faro. 

### 2.3. Hospitalization

The evaluation and characterization of hospitalization were based on the hospital admission clinical record, which provides information regarding signs and symptoms, diagnostic exams, procedures, treatments, and diagnosis on the day of discharge.

### 2.4. Blood Measurements

Serum samples were collected at baseline in fasting patients, and patients were divided according to the apelin-36 groups. Samples were centrifuged, and plasma was frozen at −80°C. At the time of sample collection for dosing apelin, other laboratorial parameters were measured. The option to analyze apelin levels by groups rather than by a continuous variable was made in order to try to better differentiate the power of lower and higher apelin levels.

The first group consisted of patients with apelin ≤98 pg/mL, group 2 included patients with apelin 98–328 pg/mL, and group 3 encompassed patients with apelin ≥329 pg/mL. In the absence of values defined for mild and moderate cardiovascular risk, the range for each apelin group was obtained from the 25th, 50th, and 75th percentiles of our sample's baseline apelin values. 

Several laboratory parameters were analyzed: albumin, hemoglobin (Hb), glycated hemoglobin (HgA1c), total cholesterol, HDL cholesterol, LDL cholesterol, triglycerides, mineral metabolism (Ca, P, and PTH), inflammation (Interleukin-6 (IL-6)), insulin resistance (homeostasis model assessment: estimated insulin resistance (HOMA-IR)), adipokines (resistin, visfatin, and adiponectin), oxidative stress (oxidized low-density lipoprotein (oxLDL)), and albumin/creatinine ratio (Table 1). To normalize the distribution of plasma visfatin, log transformation was used.

The quantification of apelin was determined using an enzyme immunoassay kit, FEIA kit (Cat. no. FEK-057-15; Phoenix Pharmaceuticals Inc., Burlingame, CA, USA), designed to detect a specific peptide and its related peptides based on the principle of “competitive” enzyme immunoassay. The immunoplate in this kit is precoated with secondary antibody, and the nonspecific binding sites are blocked. The secondary antibody can bind to the Fc fragment of the primary antibody (peptide antibody) whose Fab fragment will be competitively bound by both biotinylated peptide and peptide standard or targeted peptide in samples. The biotinylated peptide interacts with streptavidin-horseradish peroxidase (SA-HRP) which catalyzes the substrate solution. The fluorescence intensity is directly proportional to the amount of biotinylated peptide SA-HRP complex. A standard curve of known concentration can be established accordingly. The unknown concentration in samples can be determined by extrapolation to this standard curve.

The quantification of resistin and visfatin was determined by enzyme-linked immunosorbent assay using the ELISA kits (Cat. no. EK-028-36; Phoenix Pharmaceuticals Inc., Burlingame, CA, USA) and (Cat. no. EK-003-80; Phoenix Pharmaceuticals Inc., Burlingame, CA, USA), respectively. Both techniques were adapted to the Triturus automatic microplate apparatus (Grifols S.A., Barcelona, Spain). Plasma adiponectin was measured using a human adiponectin RIA Kit (Lincoln Research).

The serum levels of cholesterol, triglycerides, HDL, phosphorus, and calcium were measured using the ARCHITECT c Systems and the AEROSET System (Abbott Diagnostics Division, Abbott Laboratories Abbott Park, IL).

The low-density lipoprotein (LDL) cholesterol in human plasma was assessed using a MULTIGENT Direct LDL Assay (Abbott Diagnostics Division, Abbott laboratories Abbott Park, IL).

Serum levels of IL-6 were measured using a sandwich enzyme-linked immunoassay (ELISA) kit (eBioscience, San Diego, CA, USA), and OxLDL was measured using an immunoenzymatic assay.

Hemoglobin, glycated hemoglobin, and PTH levels were measured using a spectrophotometry technique, a high-performance liquid chromatography (HPLC), and electrochemiluminescent immunoassay (ECLIA), respectively. 

### 2.5. Insulin Resistance Assessment

The degree of insulin resistance was estimated using the homeostasis model assessment (HOMA-IR) described by Matthews et al. [10]. HOMA was calculated using the following formula:

(1) HOMA-IR(mmol/L×μU/mL)   =fasting  glucose(mmol/L)  ×fasting  insulin(μU/mL)/22.5.

### 2.6. Renal Function Assessment

This analysis included all participants with creatinine measures at baseline. Serum creatinine was measured by the enzymatic method, using the ARCHITECT device (Abbott Diagnostics Division, Abbott Laboratories Abbott Park, IL). GFR was estimated using a formula derived by the modification of diet in renal disease study group as follows [11]:

(2) eGFR=186.3×(serum  creatinine)−1.154×age−0.203 ×(0.742  if  female)×(1.21  if  black).

### 2.7. Echocardiography

Transthoracic echocardiography was performed using a General Electrical Medical Systems Echograph, model Vivid 7 with a probe (GE Healthcare, Wisconsin, USA). Data were recorded on computer and film and were analyzed always by the same technician.

### 2.8. Carotid Echodoppler

Carotid echodoppler was performed using a General Electrical Medical Systems Echograph, model Vivid 4 with a linear probe of 10 MHz (GE Healthcare, Wisconsin, USA). For the assessment of the carotid artery intima-media thickness (IMT), the protocol of the American Society of Echocardiography was followed. Data were recorded and analyzed by the same technician. 

### 2.9. Definitions

Left ventricular mass index (LVMI) was calculated by applying the regression equation from Penn convention:

(3) LV  mass=1,04([LVIDD+PWTD+IVSTD]3    −[LVIDD]3)−13,6 g,

where LVIDD is the left ventricular internal diameter in diastole, PWTD is the posterior wall thickness in diastole, and IVSRD is the interventricular septum thickness in diastole. LVMI was then obtained by dividing LV mass by body surface area.

PP was considered as the difference between systolic and diastolic pressures:

(4) $$\begin{array}{c} \text{PP}=\text{systolic pressure}-\text{diastolic pressure.} \end{array}$$

Increased cardiovascular risk was considered for PP values greater than 50 mmHg and IMT > 0.9.

### 2.10. Outcomes

The primary outcome of this study was cardiovascular mortality. Deaths were confirmed by review of autopsy reports, death certificates, medical records, or information obtained from the next of kin or family members and classified, according to its cause, as cardiovascular or noncardiovascular death. Cardiovascular deaths were defined as mortality caused by coronary heart disease, heart failure, peripheral vascular disease, and cerebrovascular disease.

Secondary outcomes comprised hospitalization due to cardiovascular causes and the assessment of any possible relationship between apelin and renal disease.

Hospitalization days were also classified as due to cardiovascular causes or due to noncardiovascular causes. Hospitalization days due to cardiovascular causes which met the same criteria for cardiovascular deaths but with no mortality. Progression of renal failure was assessed during the follow-up consultations by performing analytical evaluations of renal function (measurement of creatinine and estimation of GFR). Patients with an eGFR < 10 mL/min started a depurative technique (DT), hemodialysis, or peritoneal dialysis according to patient's choice.

### 2.11. Statistical Analyses

We used descriptive statistics, and for comparison between groups we used the ANOVA test. The Kaplan-Meier method for measuring patient survival rate was applied, and a comparison between the three tertiles was based on the log rank test. The risk factors and their hazard ratio (HR) were calculated using a backward stepwise likelihood ratio (LR) Cox regression for mortality and a multivariate logistic regression for hospitalization. Simple linear regressions and Pearson correlations were used to investigate any possible correlation between apelin and renal disease and between apelin and cardiovascular risk factors. Differences were considered statistically significant for *P* values < 0.05. Statistical analyses were performed using the SPSS program, v17.0. 

The study was submitted to and approved by the administration and ethics committee. The study was conducted according to the principles of the Declaration of Helsinki. Study procedures were only performed after signing the informed consent.

## 3. Results

A total of 150 patients were evaluated during a 87-month period from January 2005 to December 2011. The mean age of these patients was 62.67 ± 10.95 years (42–87), and 38.7% of them (58) were female. The mean follow-up time was 35.7 ± 15.3 months (8–87). 

In terms of allocation of patients into groups according to their serum apelin-36 values and following the definition of the 25th, 50th, and 75th percentiles, all three groups encompassed a total of 50 patients. All the assessed variables presented significant differences between the three apelin level groups (Table 2). 

As presented in Table 2, patients of group 3 revealed higher adiponectin levels and greater eGFR, as well as lower values of resistin, log visfatin, IL-6, OxLDL, HOMA-IR, LVMI, PP, IMT, PTH, and phosphorus. 

**Figure 1:**
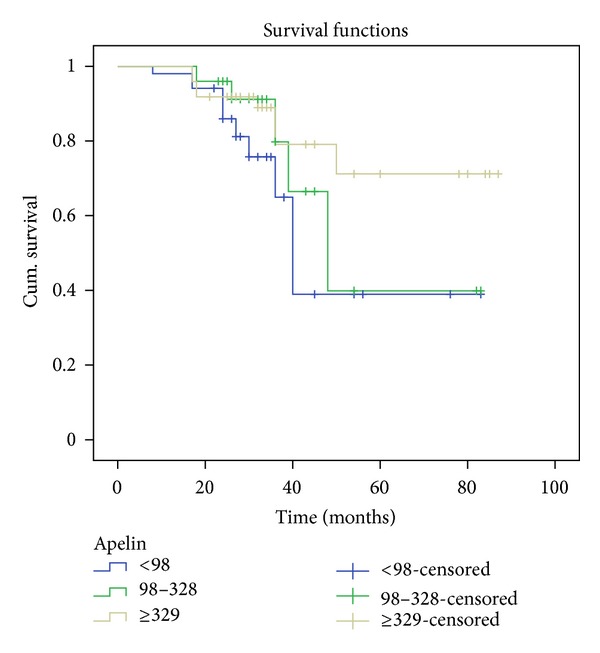
Kaplan-Meier survival analysis.

Using the Kaplan-Meier analysis, it was observed that patients' survival at 83 months was 39% in group 1, 40% in group 2, and 71.2% in group 3 (Figure 1). The log rank test confirmed the existence of significant differences between the three groups (log rank = 6.413; *P* = 0.046).

Variables such as age, sex, apelin-36 levels, HOMA-IR, adiponectin, IL-6, phosphorus, PTH, eGFR, creatinine, Hb, OxLDL, resistin, and visfatin were analyzed using a multivariate Cox regression to identify independent risk factors of mortality. Age, eGFR, and apelin-36 levels were found to predict patient survival (*P* < 0.05) in opposition to sex, HOMA-IR, adiponectin, IL-6, phosphorus, PTH, creatinine, Hb, OxLDL, resistin and visfatin (*P* > 0.05) (Table 3). A statistically significant decrease in mortality was observed with apelin levels ≥329 pg/mL (Table 4). 

Regarding cardiovascular hospitalization, patients in group 1 presented a greater mean length (15.2 ± 15.8 days (0–79)) when compared with patients in group 2 (4.7 ± 7.4 days (0–34)) and group 3 (0.9 ± 2.9 days (0–14)). These differences between groups were statistically significant (*P* < 0.001). 

In order to identify independent risk factors for hospitalization, variables such as age, sex, apelin, adiponectin, IL-6, eGFR, creatinine, LVMI, OxLDL, resistin, and visfatin were analyzed through a multivariate logistic regression. Creatinine, eGFR, and the adipokines apelin, resistin, and visfatin were found to predict the need for hospitalization (*P* < 0.05) in opposition to the other assessed variables (*P* > 0.05) (Table 5). A statistically significant decrease in the risk of hospitalization was observed with apelin levels ≥329 pg/mL (Table 6).

The association of apelin with renal disease and cardiovascular risk factors was also assessed. By applying simple linear regressions, serum apelin levels were found to be negatively correlated with IMVE (*R* = −0.588, *P* = 0.0001), creatinine (*R* = − 0.316, *P* = 0.0001), IL-6 (*R* = −0.708, *P* = 0.0001), IMT (*R* = −0.621, *P* = 0.0001), systolic BP (*R* = −0.441, *P* = 0.0001), PP (*R* = −0.588, *P* = 0.0001), and OxLDL (*R* = −0.669, *P* = 0.0001), while it was positively correlated with eGFR (*R* = 0.357, *P* = 0.0001). These results were confirmed by Pearson correlations. 

Finally, we also investigated the association of apelin levels and the progression of renal disease. It was observed that age and levels of apelin and phosphorus were independent predictors of patients' renal disease progression to a DT (*P* < 0.05). There was also a statistically significant decrease in the risk of progression to a DT with apelin levels >329 pg/mL (Table 7).

## 4. Conclusions

We evaluated the association of apelin levels with mortality and hospitalization events, as well as the relationship of apelin with renal function and cardiovascular risk factors, in a homogeneous population of type 2 diabetic patients with a diagnosis of mild to moderate CKD.

The mortality data collected revealed significant differences among the three apelin groups. Patients with higher apelin levels tended to have better survival rates than patients with lower levels of apelin. Furthermore, using Cox analysis, apelin was found to independently predict patient survival, with a decreased risk of cardiovascular mortality associated with higher apelin levels. All three groups of apelin presented established cardiovascular risk factors, so we believe there is an unbalance that leads to an increased production of activating factors (e.g., resistin, visfatin, PTH, and phosphorus) and a decreased production of protective factors (e.g., apelin) that may explain these results.

The results of our survival functions demonstrated that lower apelin levels are predictive of cardiovascular mortality in type 2 diabetic patients with a diagnosis of mild to moderate CKD. To our knowledge, although the association of apelin and the cardiorenal axis is not new, so far there are no published studies that corroborate this association between apelin and cardiovascular mortality. Thus, our results raise interest for further analysis assessing apelin as a possible marker/predictor of cardiovascular mortality in this population.

Similar results were obtained when assessing cardiovascular hospitalization and progression of renal disease in this population. Multivariate logistic regression analysis presented apelin as a predictive variable of the need for cardiovascular hospitalization and for progression of renal disease to a DT. Lower apelin levels were associated with increased risks of cardiovascular hospitalization and disease progression.

We also approached the association of apelin levels with renal disease and cardiovascular risk factors. Our results suggested that apelin levels were inversely correlated with well-known cardiovascular risk factors such as LVMI, IMT, systolic BP, PP, and OxLDL and directly correlated with the renal function parameter eGFR. Thus, it seems that, as apelin increases, renal function improves and cardiovascular risk decreases. This data seems to be in accordance with a previously reported study [12] that has already correlated apelin with some echocardiographic features and inflammatory markers in hemodialyzed patients. Interestingly, a recent study by Leal et al. [13], analyzing apelin plasma levels in hemodialyzed patients, refuted that apelin could possibly be associated with cardiovascular risk in hemodialyzed patients. Further studies with greater sample size and robust statistical analysis are necessary in order to better inquire the association between apelin and cardiovascular disease pathogenesis and risk. It would be particularly interesting to conduct further studies in earlier stages of renal disease since we present, in opposition to the other mentioned studies, results of patients that were not undergoing any DT at the beginning of the study.

In conclusion, our results suggest that, among the analyzed variables, apelin, age, and eGFR are the main predictors of cardiovascular mortality, and apelin, creatinine, resistin, and visfatin are the main predictors of hospitalization in type 2 CKD diabetic patients. Our results also tend to show that the lower the level of apelin, the greater the risk of cardiovascular mortality and hospitalization. In addition, it was observed that apelin seems to be associated with the progression of renal disease to a DT, with patients in the group of lower apelin levels being more likely to start a depurative technique. This might be possibly associated with a potential protective role of apelin; however, further studies are necessary in order to clearly define this association. Finally, we have also found that apelin levels are negatively correlated with well-known cardiovascular risk factors and positively correlated with eGFR. This fact enhances the possibility of a cardiovascular and renal protective role of apelin. These results demonstrate that apelin levels might have a significant clinical use as a marker/predictor of cardiovascular mortality and need for hospitalization, and a therapeutic role for CKD patients with cardiovascular disease is plausible.

Despite the relatively small sample studies and the limited statistical power of these analyses, features that can be pointed as the main limitations of this paper, these are preliminary results of a long-term project, and future stronger data will be presented. However, these initial results already present new important insights regarding the association of apelin and cardiovascular mortality, as we believe that this is the first paper presenting apelin as a predictor of mortality in diabetic patients with mild to moderate CKD.

We believe further studies are required in order to confirm these associations, particularly to assess a possible cardioprotector role of apelin, in earlier stages of renal disease, as well as to clarify how it interacts with other nontraditional cardiovascular risk factors on renal disease.

## Figures and Tables

**Table 1:** Patients' baseline characteristics.

Characteristics	Values
Number of patients enrolled, *n*	150
Age (years)	62.67 ± 10.95
Gender, M/F (%)	92/58 (61.3/38.7)
BMI (Kg/m^2^)	26.3 ± 2.7
Blood pressure (mmHg)	130.9/77.4 ± 18.1/10.9
Hb (g/dL)	12.6 ± 1.9
HgA1c (%)	7.5 ± 1.5
Total cholesterol (mg/dL)	192.7 ± 40.1
Triglycerides (mg/dL)	154.8 ± 88.9
PTH (pg/mL)	138.9 ± 50.8
Phosphorus (mg/dL)	4.3 ± 1.2
Calcium (mg/dL)	9.4 ± 0.9
Creatinine (mg/dL)	1.9 ± 1.0
Albumin/creatinine ratio (*μ*g/mg)	244.9 ± 116.0
eGFR (mL/min)	42.7 ± 23.6
Albumin (g/dL)	4.1 ± 0.6
HDL (mg/dL)	50.4 ± 20.8
LDL (mg/dL)	113.0 ± 30.7
Apelin (pg/mL)	216.2 ± 45.3
IL-6 (pg/mL)	6.2 ± 3.8
Resistin (pg/mL)	6.1 ± 3.5
Visfatin (pg/mL)	62.0 ± 59.7
Adiponectin (ng/mL)	28.6 ± 8.6
OxLDL (U/L)	45.7 ± 22.8
LVMI (g/m^2^)	105.2 ± 25.7
PP (mmHg)	53.1 ± 19.3

**Table 2:** Comparison of variables among the 3 apelin-36 tertiles.

	Group 1 (<98 pg/mL) *n* = 50	Group 2 98–328 (pg/mL) *n* = 50	Group 3 ≥329 (pg/mL) *n* = 50	*P* value
Resistin (pg/mL)	1.0 ± 0.3	0.8 ± 0.6	0.5 ± 0.3	<0.001
Log visfatin (pg/mL)	2.1 ± 1.7	1.7 ± 1.7	1.3 ± 1.4	<0.001
Adiponectin (ng/mL)	10.8 ± 7.9	30.1 ± 16.5	45.7 ± 18.7	<0.001
Interleukin-6 (IL-6) (pg/mL)	8.8 ± 3.0	6.4 ± 4.0	3.3 ± 1.8	<0.001
Oxidized LDL (OxLDL) (U/L)	67.3 ± 18.7	39.0 ± 19.0	30.1 ± 9.1	<0.001
Estimated glomerular filtration rate (eGFR) (mL/min)	29.7 ± 20.3	48.6 ± 21.2	49.5 ± 24.1	<0.001
Homeostasis model of assessment: insulin resistance (HOMA-IR)	3.8 ± 1.3	2.1 ± 1.5	0.6 ± 0.4	<0.001
Parathyroid hormone (PTH) (pg/mL)	217.4 ± 135.0	120.2 ± 61.4	76.3 ± 41.2	<0.001
Phosphorus (mg/dL)	5.2 ± 1.4	4.3 ± 0.8	3.6 ± 0.7	<0.001
Albumin/creatinine ratio	268.9 ± 108.6	255.5 ± 123.8	209.2 ± 108.7	0.026
Left ventricular mass index (LVMI) (g/m^2^)	133.2 ± 16.2	100.5 ± 15.3	80.8 ± 9.1	<0.001
Intima-media thickness (IMT) (mm)	1.2 ± 0.2	1.0 ± 0.3	0.8 ± 0.1	<0.001
Pulse pressure (PP) (mmHg)	68.7 ± 13.9	47.0 ± 19.3	43.1 ± 12.3	<0.001

**Table 3:** Multivariate Cox regression analysis results for cardiovascular mortality.

	Adjusted HR (95% CI)	*P* value
Age	1.013 (1.103–2.107)	0.016
Sex	1.088 (0.251–4.718)	0.910
Apelin	0.981 (0.967–0.996)	0.012
HOMA-IR	0.987 (0.922–1.901)	0.968
Adiponectin	0.885 (0.786–0.996)	0.742
IL-6	1.124 (0.881–1.434)	0.349
Phosphorus	1.585 (0.969–2.594)	0.067
PTH	1.000 (0.992–1.007)	0.922
eGFR	0.886 (0.790–0.997)	0.043
Creatinine	1.152 (1.138–1.937)	0.328
Hb	1.701 (1.103–2.625)	0.216
OxLDL	1.063 (0.995–1.136)	0.071
Resistin	0.674 (1.458–6.679)	0.423
Visfatin	0.654 (1.106–3.724)	0.347

**Table 4:** Mortality and Cox regression results by group of apelin.

Apelin group (pg/mL)	No. of patients	No. of deaths	Adjusted HR (95% CI)
≤98	50	17	Reference
98–329	50	14	0.388 (0.078–1.345)
≥329	50	4	0.026 (0.013–0.954)

**Table 5:** Multivariate logistic regression analysis results for cardiovascular hospitalization.

	Adjusted OR (95% CI)	*P* value
Age	0.974 (0.912–1.040)	0.434
Sex	0.852 (0.218–3.330)	0.818
Apelin	0.548 (0.302–0.817)	0.004
Adiponectin	0.941 (0.879–1.008)	0.083
IL-6	1.017 (0.800–1.294)	0.889
eGFR	0.983 (0.940–1.029)	0.464
Creatinine	1.095 (1.066–1.144)	0.037
LVMI	1.024 (0.970–1.081)	0.386
OxLDL	1.056 (0.995–1.122)	0.072
Resistin	1.097 (0.992–1.192)	0.043
Visfatin	1.035 (1.008–1.061)	0.009

**Table 6:** Hospitalization and logistic regression results by group of apelin.

Apelin group (pg/mL)	No. of patients	No. of patients hospitalized	Adjusted OR (95% CI)
≤98	50	35	Reference
98–329	50	29	0.667 (0.558–1.748)
≥329	50	9	0.615 (0.439–1.319)

**Table 7:** Depurative technique and logistic regression results by group of apelin.

Apelin group (pg/mL)	No. of patients	No. of patients starting a DT	Adjusted OR (95% CI)
≤98	50	20	Reference
98–329	50	10	1.500 (0.520–4.323)
≥329	50	7	0.871 (0.456–1.492)
